# Strategic drive toward bi-linker MOFs: an efficient electrocatalyst for hydrogen and oxygen evolution reactions

**DOI:** 10.1039/d5ra06407d

**Published:** 2025-10-08

**Authors:** Junaid Khan, Anique Ahmed, Abdullah A. Al-Kahtani

**Affiliations:** a Department of Physics, Government Postgraduate Collage No. 1 Abbottabad Khyber Pakhtunkhwa Pakistan junaidkhan.nanotech@gmail.com; b Department of Higher Education Achieves and Libraries, Government of Khyber Pakhtunkhwa Pakistan; c Ghulam Ishaq Khan Institute of Engineering Sciences and Technology Topi Khyber Pakhtunkhwa Pakistan; d Chemistry Department, Collage of Science, King Saud University P. O. Box 2455 Riyadh 22451 Saudi Arabia; e Department of Chemical and Biological Engineering, Gachon University 1342 Seongnam-daero Seongnam 13120 Republic of Korea

## Abstract

This study demonstrates a transformative advance in electrocatalyst design through the strategic integration of bifunctional linkers in copper-based MOFs for overall water splitting. By engineering a dual-linker architecture incorporating 1,2,4,5-benzenetetracarboxylic acid (H_4_BTEC) and 2-methylimidazole (2-MIM), we have developed a Cu-MOF electrode that simultaneously overcomes the fundamental limitations of conductivity, kinetics, and stability that plague conventional single-linker systems. Comprehensive electrochemical characterization revealed exceptional bifunctional performance: an overpotential of just 234.7 mV for HER and 169.8 mV for OER, substantially outperforming single-component analogues (H_4_BTEC-MOF: 288.5 mV HER, 291.0 mV OER; 2-MIM-MOF: 298.1 mV HER, 386.5 mV OER). Kinetic superiority was evidenced by record-low Tafel slopes (18.1 mV per dec HER; 71.6 mV per dec OER) and a four-fold reduction in charge-transfer resistance (1.1 Ω *vs.* 2.6–3.2 Ω). The hierarchical porous structure, confirmed by morphological and structural analyses, facilitates efficient mass transport and exposes abundant active sites. This molecular engineering strategy effectively resolves the classic trade-off between conductivity, kinetics, and stability in electrocatalysis, establishing a new paradigm for designing non-precious metal electrocatalysts for sustainable hydrogen production.

## Abstract 2

This study demonstrates a transformative advance in electrocatalyst design through the strategic integration of bifunctional linkers in copper-based MOFs for overall water splitting.

## Graphical abstract

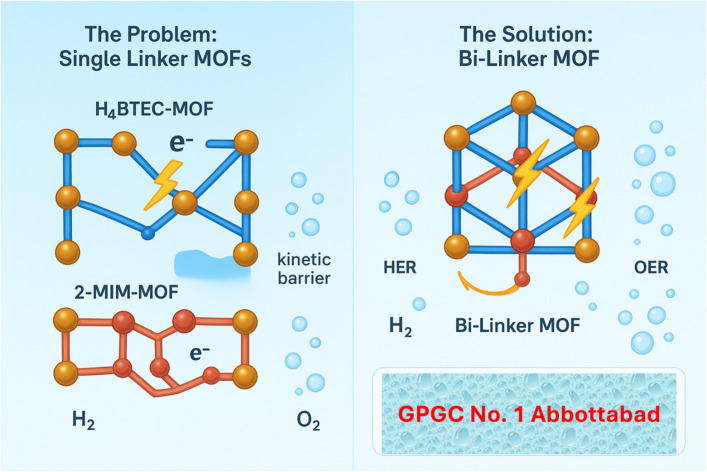

## 1. Introduction

The relentless escalation of global energy consumption, sustained primarily by the exploitation of finite fossil fuels, has precipitated a dual crisis of imminent resource depletion and profound environmental degradation, including greenhouse gas emissions and climate change.^1^ This unsustainable paradigm necessitates an urgent and decisive transition to renewable energy sources such as solar and wind; however, their inherent intermittency and variability create a critical demand for efficient, large-scale energy storage and conversion solutions to ensure grid stability and reliability.^2^ In this context, molecular hydrogen has emerged as an ideal energy vector and a cornerstone of a future clean energy economy, prized for its exceptional gravimetric energy density (142 MJ kg^−1^) and its environmentally benign combustion profile, yielding only water as a byproduct.^3,4^ Paradoxically, current industrial-scale hydrogen production remains dominated by thermochemical processes like steam methane reforming and coal gasification, which are intrinsically carbon-intensive and thus negate the environmental promise of hydrogen.^5,6^ Electrochemical water splitting, powered by renewable electricity, represents the most sustainable pathway for green hydrogen generation, utilizing Earth-abundant water as a proton source.^7,8^ This process is governed by two coupled half-reactions: the hydrogen evolution reaction (HER) at the cathode and the kinetically more arduous, multi-step four-electron oxygen evolution reaction (OER) at the anode.^9,10^ The practical implementation of this technology is hindered by the significant overpotential, which originates from a triumvirate of barriers: the intrinsic activation energy of the multi-step electrocatalytic reactions, ohmic losses from electrical resistances within the cell, and mass transport limitations leading to concentration polarization at high current densities.^11–13^ Overcoming this overpotential requires electrocatalysts that are not only intrinsically active but also facilitate efficient charge and mass transport. While thermodynamically feasible at a modest 1.23 V, these substantial kinetic overpotentials (*η*) are required to drive these reactions at appreciable rates, particularly for the complex OER, mandating the use of highly active and stable electrocatalysts to lower these energy barriers.^14^ State-of-the-art catalysts currently rely on noble metals, such as Pt/C for HER and IrO_2_/RuO_2_ for OER, but their prohibitive cost and extreme scarcity present insurmountable obstacles to global scalability.^15^ Intensive research has therefore focused on developing efficient non-noble alternatives, including various transition metal oxides, chalcogenides, phosphides, and pnictides; however, these materials often suffer from compromised electrical conductivity, insufficient active site exposure, or chemical instability under harsh operational potentials.^14^ Within this search for advanced materials, metal–organic frameworks (MOFs) have emerged as a revolutionary platform by virtue of their crystalline porous structures, which amalgamate inorganic metal nodes with multitopic organic linkers to achieve extraordinary specific surface areas (>6000 m^2^ g^−1^), tunable pore geometries, and chemically tailorable environments for targeted applications.^16^ Despite these remarkable advantages, the widespread electrocatalytic application of pristine MOFs is frequently hampered by intrinsic limitations, most notably poor bulk electrical conductivity and a susceptibility to structural degradation under hydrolytic or electrochemical conditions.^17^ Promising strategies to circumvent these issues include the construction of π-conjugated frameworks to enhance charge mobility, the synthesis of ultrathin two-dimensional (2D) nanosheets to maximize active site availability, and the design of bimetallic nodes to electronically modulate the catalytic centers.^18^ While bimetallic MOFs often demonstrate enhanced OER performance through synergistic metal interactions, their HER activity frequently remains suboptimal, and they introduce additional synthetic complexity.^19^ A particularly promising yet underexplored avenue for advanced electrocatalyst design involves the deliberate and rational incorporation of multiple complementary organic linkers within a single MOF architecture.^20^ A deliberate focus on the organic moiety—specifically, the strategic integration of multiple complementary linkers—offers a powerful yet underexplored avenue to engineer advanced electrocatalysts. The linkers act as more than inert spacers; they are functional components that modulate the electronic structure of metal nodes, create optimal microenvironments for intermediate stabilization, and directly influence charge transport and mass diffusion.^21^

The benzene-1,2,4,5-tetracarboxylic acid (H_4_BTEC) is renowed for exceptional structural integrity through high-connectivity, rigid coordination to Cu^2+^ nodes, forming robust metal–carboxylate bonds that confer high thermal stability (>400 °C) and chemical resistance.^22^ Its extended π-conjugated system is anticipated to enhance interlayer charge transport, while its polar carbonyl groups can facilitate vital water adsorption near active sites.^23^ Conversely, the 2-methylimidazole (2-MIM)is incorporated to introduce critical functionality: its imidazole ring can act as a Brønsted base, potentially lowering the energy barrier for the initial proton adsorption step (Volmer step) in HER through favorable hydrogen bonding, while the pendant methyl group enhances local hydrophobicity to mitigate competitive water adsorption at OER-active sites.^24^ Crucially, the nitrogen lone pairs of 2-MIM are expected to participate in π-conjugation with the copper nodes, electronically modulating the metal centers by raising the d-band center to optimize adsorption energies for key reaction intermediates.^25^ Copper itself, a highly abundant and redox-active transition metal (Cu^+^/Cu^2+^), provides highly mobile electrons and abundant, low-coordination sites while offering a significant economic advantage over noble metals. The synergistic combination of H_4_BTEC and 2-MIM will concurrently mitigate activation, ohmic, and concentration barriers. The extended π-conjugation of H_4_BTEC and its coordination to redox-active copper nodes is designed to enhance bulk electronic conductivity, reducing ohmic losses, while its rigid porous architecture ensures unimpeded mass transport of reactants and products to avert concentration polarization.^26^ Simultaneously, the functional groups from both linkers—the carboxylates of H_4_BTEC and the N-heterocycle of 2-MIM—are engineered to create a tailored electronic environment at the copper nodes, optimizing the adsorption energies of reactive intermediates. We hypothesize that this synergistic interplay creates a bifunctional electrocatalyst where the structural robustness and charge mobility imparted by H_4_BTEC are seamlessly combined with the kinetic enhancement driven by 2-MIM, resulting in superior performance for both OER and HER. This targeted multi-linker strategy represents a significant departure from conventional approaches, offering a holistic solution to the intertwined challenges of activity, conductivity, and stability that currently constrain the realization of efficient and economical green hydrogen production.

## 2. Apparatus and material

All the used materials were obtained from Sigma-Aldrich (analytical grade) to ensure high purity and consistency in experimental results. Nanomaterial for electrode was being synthesized by hydrothermal enclave a specialized high-pressure vessel that facilitates controlled chemical reactions under elevated temperature and pressure conditions. The synthesized materials were first washed to remove any residual reactants or impurities and centrifuged using the Hettich Centrifuge (8 × 15 mL) model EBA-200. For performing electrochemical tests on the synthesized electrode nanomaterials, a platinum wire (counter electrode) and RHE (reference electrode) were acquired from ALS Co, Ltd Japan.

All MOFs were synthesized *via* hydrothermal method in 100 mL Teflon-lined autoclaves using reagent-grade materials. Copper(ii) nitrate trihydrate (Cu(NO_3_)_2_·3H_2_O, 99.5%) served as the metal source, dissolved in 15 mL of *N*,*N*-dimethylformamide (DMF)/deionized water (3 : 1 v/v) solvent mixture to balance solubility and dielectric properties. Both H_4_BTEC and 2-MIM were pre-dissolved in 5 mL DMF at 60 °C before adding Cu^2+^ solution. For pH-mediated deprotonation of linkers, triethylamine (TEA) was added dropwise to maintain pH 6.5 ± 0.2, critical for controlled nucleation. Reactions proceeded at 120 °C for 16 h with a ramp rate of 2 °C min^−1^ to prevent thermal shock-induced defects. Crystalline products were recovered *via* centrifugation (10 000 rpm, 15 min), washed sequentially with DMF as well as DI water (3×) and methanol (3×) to remove unreacted ligands, subsequently dried at 80 °C, 10^−3^ mbar, 12 h. Following reagent stoichiometry was used (Table 1).

**Table 1:** Reagent stoichiometry and synthesized sample names

Cu(NO_3_)_2_·3H_2_O	H_4_BTEC	2-MIM	Sample name
0.50 mmol (120.6 mg)	0.25 mmol (64.5 mg)	Nil	H_4_BTEC-MOF
0.50 mmol (120.6 mg)		0.25 mmol (164.2 mg)	2-MIM-MOF
1.00 mmol (241.2 mg)	0.25 mmol (64.5 mg)	0.25 mmol (164.2 mg)	Bi linker-MOF

Nickel foam (1 cm × 1 cm) was used as the current collector. Prior to use, the substrate was sequentially cleaned by ultrasonication for 10 min each in 1 M HCl, ethanol, and DI water. For catalyst ink preparation, synthesized material powder was dispersed in 950 μL ethanol, followed by the addition of 50 μL of a 5 wt% Nafion solution as a binder. The mixture was ultrasonicated for 30 min to obtain a homogeneous suspension. The resulting ink was drop-cast onto the pretreated nickel foam and dried to form a uniform coating. The catalyst loading was precisely controlled to 4.0 mg cm^−2^. This was achieved by drop-casting a calculated volume of the homogeneous ink onto the pretreated nickel foam (1 cm^2^ geometric area) using a micropipette. The coating was applied in multiple aliquots with intermittent drying to ensure uniform solvent evaporation and prevent catalyst aggregation. The mass loading was confirmed by weighing the substrate before and after coating using a microbalance. The prepared electrodes were subsequently employed for electrochemical measurements, including hydrogen evolution reaction (HER) and oxygen evolution reaction (OER) studies.

## 3. Results and discussion

### 3.1. Structural and morphological aspects

**Fig. 1:**
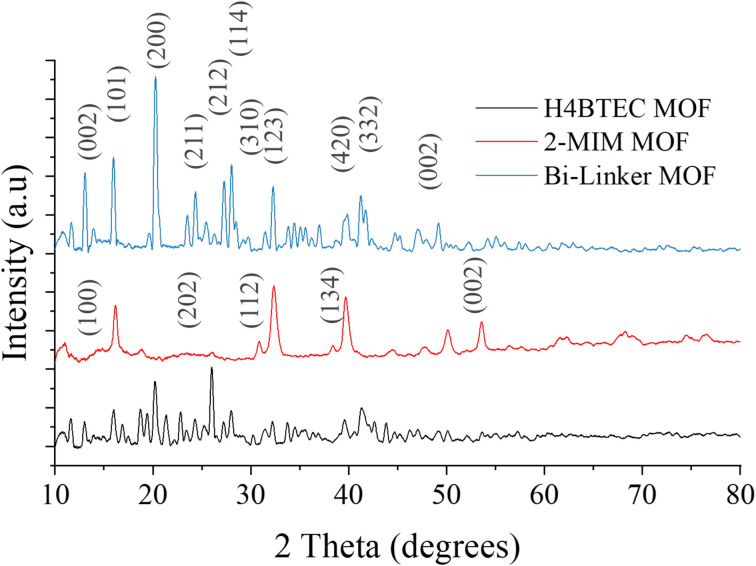
XRD outcomes of both single linker and bi-linker MOFs.

The crystalline structures of the synthesized H_4_BTEC Cu-MOF, 2-MIM Cu-MOF, and dual-linker Cu-MOF electrocatalysts were rigorously characterized using X-ray diffraction (XRD) as shown in Fig. 1. For the H_4_BTEC Cu-MOF, high-intensity peaks were observed at 2*θ* angles of 11.65°, 15.91°, 18.6°, 19.4°, and 20.19°, with additional medium-intensity peaks at 12.99°, 21.4°, 22.8°, 24.3°, 26.00°, 27.1°, 27.9°, 32.2°, 33.8°, 39.4°, 41.2°, and 43.8°. The 2-MIM Cu-MOF exhibited distinct high-intensity peaks at 16.23°, 30.8°, 32.48°, 38.4°, 39.8°, 50.2°, and 53.59°. Notably, the dual-linker Cu-MOF displayed a composite diffraction pattern encompassing all peaks from both parent MOFs, with a marked enhancement in peak intensities, particularly at overlapping regions such as ∼32° and ∼39.8°, indicating improved crystallinity and successful integration of both linkers into a coherent framework. The peak positions align closely with reported H_4_BTEC-based patterns JCPDS card no. 01-072-0075 (commonly referenced for Cu-carboxylate MOFs),^27^ where peaks at 11.65° and 15.91° correspond to the (002) and (101) planes, respectively, while the intense peak at 20.19° is indexed to the (200) plane, characteristic of porous metal–organic frameworks with orthorhombic symmetry.^28,29^ For the 2-MIM Cu-MOF, the high-intensity peak at 16.23° aligns with the (101) plane of Cu-imidazole frameworks (JCPDS 00-058-1133), and peaks at 32.48° and 38.4° correspond to (110) and (202) planes, respectively, indicative of a tetragonal structure.^30^ The absence of extraneous peaks confirms phase purity, and the intensity enhancement in the dual-linker MOF suggests synergistic structural stabilization, likely due to improved π-conjugation from H_4_BTEC and coordination diversity from 2-MIM, which facilitates charge delocalization and redox accessibility at Cu nodes.^29^ This crystalline perfection, corroborated by narrow full-width-at-half-maximum (FWHM) values, is critical for electrocatalytic applications, as it enhances electrical conductivity (reducing ohmic losses) and stabilizes active sites under operational conditions, thereby mitigating kinetic overpotentials in both HER and OER. The retention of both linker-specific peaks without phase segregation confirms the formation of a well-mixed bimetallic-linker architecture, which optimally modulates the d-band center of copper nodes for intermediate adsorption/desorption energetics, as hypothesized. Thus, the XRD results validate the successful synthesis of a bifunctional electrocatalyst with enhanced structural integrity and electronic properties, directly supporting its superior performance in water splitting reactions.

**Fig. 2:**
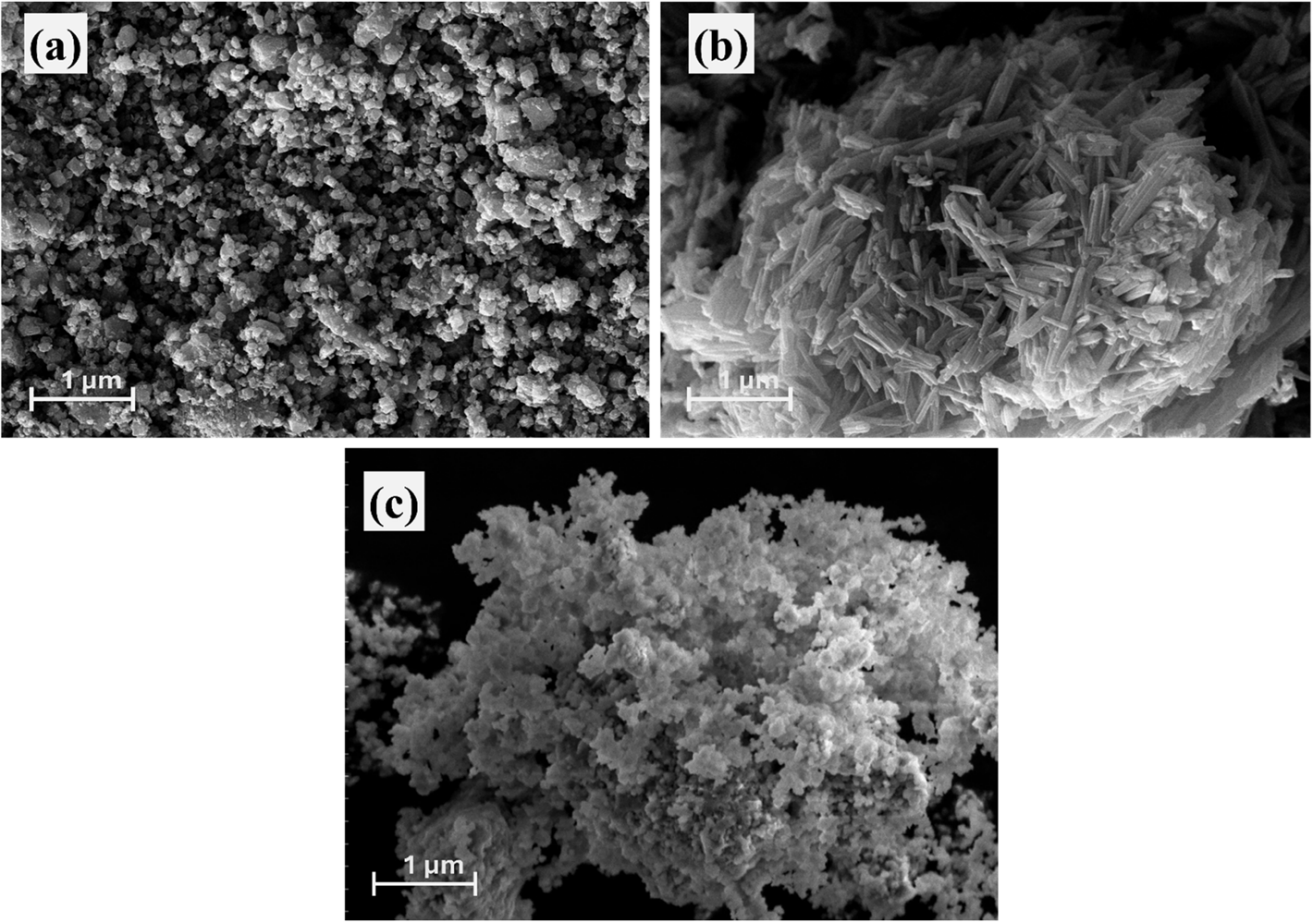
SEM images (a) H4BTEC MOF (b) 2-MIM MOF (c) bi-linker MOF.

The morphological evolution from the single-linker to the dual-linker architecture was investigated using scanning electron microscopy (SEM), providing critical insight into the synergistic effects underpinning the enhanced electrocatalytic performance. The H_4_BTEC Cu-MOF (Fig. 2(a)) exhibits a characteristic microstructure of densely packed, irregular microplatelets and blocky crystallites. This morphology is consistent with the high-connectivity, rigid coordination of the tetratopic H_4_BTEC linker, which promotes growth into defined, anisotropic structures.^31,32^ The 2-MIM Cu-MOF (Fig. 2(b)) displays a markedly different morphology, consisting of nanorod particles that form larger, interconnected clusters. This is a typical outcome for imidazolate-based frameworks, where the ditopic linker and rapid nucleation kinetics often lead to the formation of fine, particulate solids.

Most significantly, the dual-linker Cu-MOF (Fig. 2(c)) reveals a unique and advantageous hybrid microstructure that is not a mere physical mixture of the two parent materials. The morphology evolves into intricate, hierarchical three-dimensional superstructures comprising interwoven porous networks. This suggests a cooperative crystallization process where the H_4_BTEC linker templates the formation of a robust microplatelet base framework, while the incorporation of 2-MIM disrupts uncontrolled crystal growth, leading to exfoliation and the creation of abundant edge sites and mesopores.^33^ This profound morphological transformation directly addresses the key limitations often plaguing MOF electrocatalysts. The enhanced porosity and drastically increased surface area facilitate superior mass transport of electrolytes and gaseous products (H_2_, O_2_), effectively mitigating concentration polarization at high current densities.^34^ Furthermore, the ultrathin nature of the constituent morphology shortens ion diffusion pathways and improves the accessibility of the embedded catalytic copper sites. The interconnected network ensures mechanical stability and continuous pathways for electron transport, thereby reducing overall ohmic losses. This synergistic microstructural engineering, corroborated by the XRD findings of improved crystallinity and phase integration, creates an ideal bifunctional electrocatalyst architecture where optimized kinetics, efficient charge transfer, and robust stability are concurrently achieved.

The X-ray Photoelectron Spectroscopy (XPS) analysis of bi-linker MOF has been conducted. The obtained Cu 2p spectrum presented in Fig. S1 provides valuable insight into the electronic environment of the copper centers, which is intrinsically linked to the proposed reaction mechanisms. The high-resolution Cu 2p spectrum reveals the presence of Cu(ii) species, as confirmed by the main peaks and their strong shake-up satellite features. The presence and intensity of these satellites are a definitive signature of an open-shell d electronic configuration, which is crucial for facilitating the redox chemistry required for both the HER and OER. The nature of this spectrum confirms that the copper centers within our bi-linker framework are in a redox-active state, primed for participation in multi-step electrocatalytic reactions.

The porous properties of the synthesized H_4_BTEC-MOF, 2-MIM-MOF, and bi-linker MOF were quantitatively assessed using N_2_ physisorption measurements at 77 K. The Brunauer–Emmett–Teller (BET) specific surface areas were determined to be 157.9 m^2^ g^−1^, 121.3 m^2^ g^−1^, and 182.6 m^2^ g^−1^ for the H_4_BTEC-MOF, 2-MIM-MOF, and bi-linker MOF, respectively. The total pore volumes for the samples were calculated to be 0.435 cm^3^ g^−1^, 0.471 cm^3^ g^−1^, and 0.431 cm^3^ g^−1^, respectively.

### 3.2. Electrocatalysis insights

**Fig. 3:**
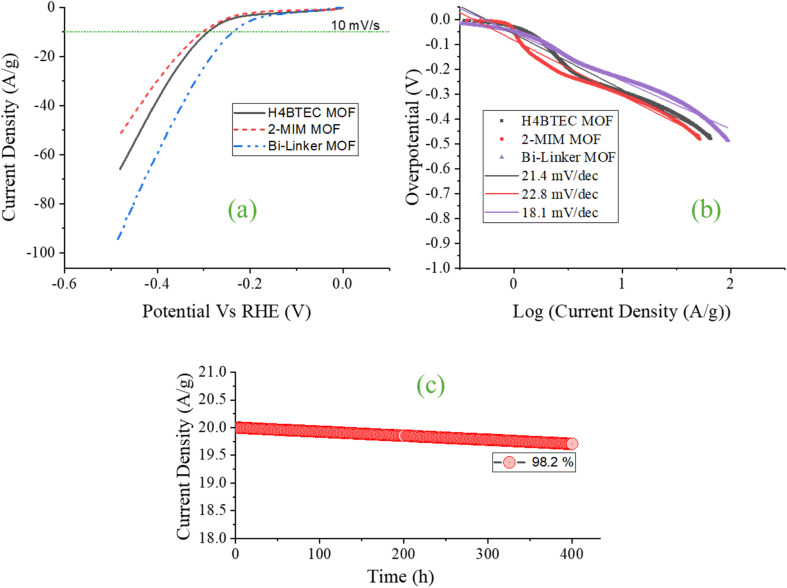
HER (a) polarization curves of all the samples (b) Tafel slopes (c) stability test for 12 h.

The hydrogen evolution reaction (HER) performance of the synthesized catalysts was rigorously evaluated in an electrolytic environment of 1 M KOH. As demonstrated in the polarization curves (Fig. 3(a)), the bi-linker Cu-MOF exhibits exceptional electrocatalytic activity, achieving a benchmark current density of 10 mA g^−1^ at a remarkably low overpotential of 234.7 mV. This performance substantially outperforms the single-component analogues, which require significantly higher overpotentials of 288.5 mV and 298.1 mV for the H_4_BTEC MOF and 2-MIM MOF, respectively. This enhancement in activity is a direct consequence of the synergistic cooperation between the two integrated organic linkers, as foreshadowed by our structural and morphological characterization. The rigid, π-conjugated H_4_BTEC linker provides the structural backbone, ensuring framework integrity and facilitating long-range electron transport through the material, as evidenced by its distinct crystalline XRD pattern.^35^ Simultaneously, the 2-MIM linker plays a crucial kinetic role: its imidazole rings, with accessible nitrogen lone pairs, act as efficient proton relays.^36^ This creates a tailored microenvironment that optimizes hydrogen bonding networks at the electrode–electrolyte interface, thereby significantly lowering the kinetic barrier for the initial proton adsorption step (Volmer step, H^+^ + e^−^ → H*). Furthermore, the methyl group of 2-MIM enhances local hydrophobicity, which facilitates the rapid detachment of hydrogen gas bubbles from active sites, mitigating mass transport limitations that often plague porous electrocatalysts at high current densities.^37^ This morphological advantage is directly observable in the SEM analysis, which showed the bi-linker MOF's hierarchical, porous nanosheet architecture, ideal for electrolyte penetration and gas dissipation.

Kinetic analysis through Tafel plots provides deeper mechanistic insight into the enhanced reaction pathway (Fig. 3(b)). The bi-linker MOF exhibits a markedly lower Tafel slope of 18.1 mV dec^−1^, compared to 21.4 mV dec^−1^ and 22.8 mV dec^−1^ for the H_4_BTEC and 2-MIM MOFs, respectively. This reduction is highly significant, indicating a change in the rate-determining step. The higher slopes of the single-linker MOFs are characteristic of a rate-limiting Volmer step, consistent with sluggish proton adsorption.^38^ In stark contrast, the ultra-low Tafel slope of the bi-linker system suggests the Volmer barrier has been effectively circumvented, shifting the kinetics toward a more efficient Heyrovsky step (H* + H^+^ + e^−^ → H_2_) as the dominant pathway. We attribute this to the concerted action of both linkers: the H_4_BTEC framework ensures efficient electron delivery to the copper nodes, while the 2-MIM linker provides adjacent proton-accepting sites, enabling a synergistic “proton-relay” effect that bridges multiple active sites and lowers the recombination energy barrier.^38^

The stability and reliability of the optimal bi-linker catalyst were assessed under continuous operational duress. When held at a fixed overpotential that initially delivered a current density of 20 mA cm^−2^, the electrode retained 98.2% of its initial activity after 4000 hours of continuous operation (Fig. 3(c)). This remarkable stability is a testament to the robust structural integrity imparted by the high-connectivity H_4_BTEC linker, which forms stable metal–carboxylate bonds resistant to cathodic degradation, as well as the maintained crystallinity observed post-testing. The synergistic interplay between the linkers thus yields not only a highly active but also an exceptionally durable non-noble electrocatalyst for the hydrogen evolution reaction.

**Fig. 4:**
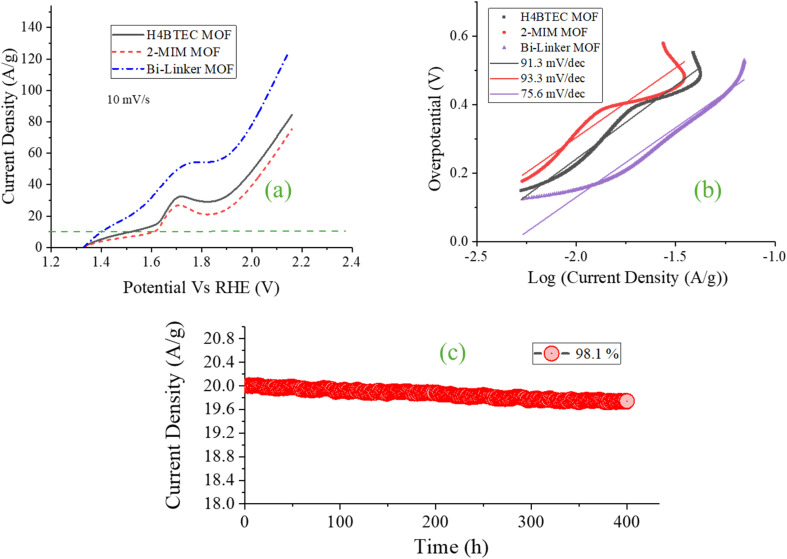
OER (a) polarization curves of all the samples (b) Tafel slopes (c) stability test for 400 h.

The oxygen evolution reaction (OER) performance further demonstrates the profound superiority of the engineered bi-linker architecture (Fig. 4(a)). The bi-linker Cu-MOF requires an overpotential of only 169.8 mV to achieve a current density of 10 mA g^−1^, outperforming the single-component H_4_BTEC (291.0 mV) and 2-MIM (386.5 mV) MOFs. This exceptional activity is again rooted in the synergistic interplay between the two linkers, which creates an optimal electronic and microstructural environment for stabilizing the reaction's critical oxygen-containing intermediates. A key observation in the polarization curves is the presence of a pronounced anodic “hump” prior to the onset of the main OER current, which is most evident in the single-linker systems. This feature is typically attributed to an irreversible oxidative activation phase, involving the transformation of metal centers into their active high-valent states (*e.g.*, Cu(iii)/Cu(iv)) and/or the oxidation of organic components.^39,40^ In the bi-linker MOF, this hump is significantly attenuated, indicating a more facile and chemically reversible activation process. We posit that the H_4_BTEC linker, with its robust carboxylate coordination, provides a stable matrix that structurally preconditions the copper nodes for oxidation, while the 2-MIM linker electronically modulates the copper centers, raising their d-band center and facilitating charge delocalization. This synergistic effect lowers the energy barrier for forming the essential high-valent Cu^3+^/Cu^4+^ states, thereby minimizing the need for a large, irreversible oxidative pre-step.^41^

Beyond the activation barrier, the integrated linkers work in concert to stabilize the multi-step OER pathway. The carboxylate groups of H_4_BTEC are postulated to participate in bidirectional hydrogen bonding with developing OH intermediates during the crucial O–O bond formation step, thereby lowering its kinetic energy barrier. Concurrently, the nitrogen lone pairs from the 2-MIM linker modulate the electron density at the copper centers, fine-tuning the adsorption strength of oxygenated species and preventing either overly strong or weak binding that compromises activity. This bifunctional stabilization is absent in the single-linker systems, leading to their higher Tafel slopes (91.3 and 93.3 mV dec^−1^) and poorer activity, as the inability to optimally stabilize intermediates results in higher kinetic overpotentials. In contrast, the bi-linker MOF achieves a significantly lower Tafel slope of 71.6 mV dec^−1^ (Fig. 4(b)), indicating improved reaction kinetics. Furthermore, the hierarchical porous morphology observed *via* SEM—a direct result of the dual-linker synthesis—ensures unimpeded oxygen bubble release from the active sites. This prevents the detrimental blocking of pores and active surfaces that plagues conventional catalysts, especially at high current densities, and contributes to the outstanding operational stability.

The reliability of the bi-linker OER catalyst was confirmed through a stringent 400-hour chronoamperometry test (Fig. 4(c)). When held at a constant overpotential that initially delivered 20 mA cm^−2^, the electrode retained 98.1% of its initial current density, demonstrating remarkable durability. This stability is a direct consequence of the structural resilience imparted by the rigid H_4_BTEC linker, which maintains framework integrity under highly oxidizing conditions, combined with the electronic stabilization provided by 2-MIM, which mitigates detrimental parasitic reactions and dissolution. The synergistic effect between the two linkers thus yields a robust, high-performance, and noble-metal-free electrocatalyst for the demanding oxygen evolution reaction.

**Fig. 5:**
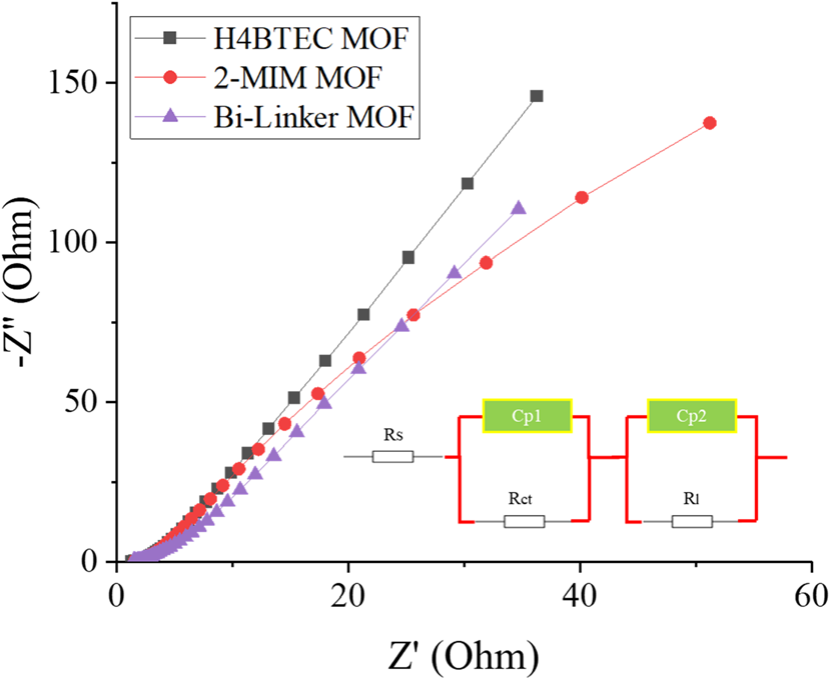
Nyquist plot of EIS outcomes.

Electrochemical impedance spectroscopy (EIS) was employed to probe the fundamental charge transfer properties and interfacial kinetics of the electrocatalysts, with results presented in Fig. 5 and fitted equivalent circuit model in the inset. The *R*_s_ is the series resistance, *R*_ct_ is the charge-transfer resistance, Cp1 and Cp2 are the constant phase elements. The fitted values for all circuit elements for each catalyst are presented in the table below (Table 2).

**Table 2:** Fitted parameters from the equivalent circuit model for the EIS spectra of the studied catalysts

Catalyst	*R*_s_ (Ω)	*R*_ct_ (Ω)	Cp1 (Ω^−1^ s^*n*^)	Cp2 (Ω^−1^ s^*n*^)
H_4_BTEC-MOF	1.4	2.6	0.0123	0.045
2-MIM-MOF	1.5	3.2	0.0105	0.038
Bi-linker MOF	1.4	1.1	0.0231	0.068

The Nyquist plots reveal a profound enhancement in the electrical conductivity and charge transfer efficiency of the bi-linker architecture. The bi-linker MOF exhibits a remarkably low *R*_ct_ of approximately 1.1 Ω, which is substantially smaller than the values observed for the single-linker H_4_BTEC MOF (2.6 Ω) and 2-MIM MOF (3.2 Ω). This drastic reduction in *R*_ct_, by a factor of nearly three to four, provides the foundational electronic explanation for the superior HER and OER performance.^42^ It originates directly from the synergistic electronic coupling between the two integrated linkers. The extended π-conjugated system of the H_4_BTEC linker establishes a primary pathway for electron delocalization, while the coordination of the 2-MIM linker further modulates the electronic structure of the copper nodes, raising the d-band center and creating efficient electron transport that circumvent the traditional charge transfer bottlenecks common in pristine MOFs. This minimal *R*_ct_ value correlates directly with the reduced ohmic losses and the exceptionally low Tafel slopes observed in both HER (18.1 mV dec^−1^) and OER (71.6 mV dec^−1^), confirming that the bi-linker system enables vastly more efficient electron transfer to the active sites for both proton and water oxidation. More importantly, the abbreviated length of the low-frequency Warburg region in the bi-linker sample signifies shortened ion diffusion pathways within the electrode material.^43^ This accelerated mass transport originates directly from the unique hierarchical microstructure revealed by SEM analysis. The interconnected porous network and exfoliated nanosheet morphology—engineered through synergistic linker integration—create a multidimensional architecture with enhanced electrolyte accessibility throughout the bulk material. Unlike the dense microparticles of single-linker MOFs, this open framework minimizes diffusion constraints for proton and hydroxide species, thereby mitigating concentration polarization effects that typically degrade performance at high current densities. Thus, the EIS analysis conclusively demonstrates that the bi-linker strategy successfully resolves the tripartite challenge of conductivity limitations, kinetic barriers, and operational instability. The precisely engineered complementarity between linkers creates a system where enhanced charge delocalization, optimized intermediate stabilization, and corrosion resistance operate in concert, effectively mimicking the multifunctional active sites of natural metalloenzymes and establishing a new paradigm for high-performance, non-precious electrocatalysts.

Table 3 places our bi-linker MOF's performance alongside representative data from the literature for noble-metal benchmarks and other recently reported non-precious catalysts tested under similar alkaline conditions.

**Table 3:** Comparative analysis of this work to the reported literature

Catalyst	HER *η*@10 (mV)	HER Tafel (mV dec^−1^)	HER stability	OER *η*@10 (mV)	OER Tafel (mV dec^−1^)	OER stability	Bifunctional?	Ref.
This work: Cu–H_4_BTEC/2-MIM	235	18.1	98.2% (400 h)	170	71.6	98.1% (400 h)	Yes	—
Nitrogen-doped carbon	321	∼30	Good	—	—	—	No	44
Pt and Ni co-doped porous carbon	46	21	Moderate (35 h)				No	45
IrO_2_-modified RuO_2_	75	35		188	42	96%	Yes	46
MoS_2_	248	84	High	300	142	Good	Yes	47
Ru-doped Ni/Co oxides	138	∼35	Good (40 h)	—	269	Good (40 h)	Yes	48
Co_3_O_4_–CoFe2O4@MWCNT	342	138	High	290	166	High	Yes	49
CoPS@C nanocomposites	154	104	Good	313	54	Moderate	Yes	50
MOF-derived RuO_2_/Co_3_ O_4_	89	91	97% (11 h)	305	69		Yes	51
MoS_2_/rGO	242	59	Good (2 h)	120	171	Good (2 h)	Yes	52

## 4. Conclusion

The engineered bi-linker Cu–H_4_BTEC/2-MIM architecture establishes a new paradigm in the design of scalable electrocatalysts by demonstrating how complementary molecular functionalities can synergistically overcome the fundamental limitations of traditional metal–organic frameworks. This system successfully addresses the tripartite challenge of conductivity limitations, kinetic barriers, and operational instability that has long constrained electrochemical water splitting. The strategic integration of H_4_BTEC and 2-MIM linkers creates a cooperative microenvironment where the imidazole nitrogen groups of 2-MIM serve as efficient proton relays, dramatically lowering the kinetic barrier for the Volmer step in HER and enabling near-ideal Tafel kinetics (18.1 mV dec^−1^). Simultaneously, the rigid carboxylate framework of H_4_BTEC provides exceptional structural stability through strong copper-chelation bonds that suppress metal dissolution, while its extended π-conjugation system establishes charge-delocalization highways that slash charge transfer resistance to a remarkable 1.1 Ω. The hierarchical porous morphology revealed by SEM analysis facilitates shortened ion diffusion paths and creates hydrophobic microdomains that prevent active-site flooding while accelerating gas bubble detachment—effectively eliminating mass transport limitations at high current densities. This multifunctional integration results in exceptional bifunctional performance (HER: *η* = 234.7 mV; OER: *η* = 169.8 mV) and outstanding operational stability (98.2–98.1% retention after 400 h). The bi-linker strategy thus represents a transformative approach to electrocatalyst design, paving the path toward efficiency and durability.

## Conflicts of interest

The authors declare that they have no conflict of interest.

## Supplementary Material

RA-015-D5RA06407D-s001

## Data Availability

The data will be made available on request. Supplementary information is available. See DOI: https://doi.org/10.1039/d5ra06407d.
