# Supplementary material for: Strategic drive toward bi-linker MOFs: an efficient electrocatalyst for hydrogen and oxygen evolution reactions
Source: RSC Adv. 2025 Oct 8;15(44):37361–70. doi: 10.1039/d5ra06407d (PMC12506645; doi:10.1039/d5ra06407d)

Supplementary Information (SI) for RSC Advances.
This journal is © The Royal Society of Chemistry 2025

Strategic Drive Toward Bi-Linker MOFs: An Efficient Electrocatalyst for Hydrogen and Oxygen Evolution Reactions

Junaid Khan[a, b **], Anique Ahmed[c], Abdullah A. Al-Kahtani[d]

[a]Department of Physics, Government Postgraduate Collage No.1, Abbottabad, Khyber Pakhtunkhwa, Pakistan

[b]Department Of Higher Education Achieves and Libraries, Government of Khyber Pakhtunkhwa, Pakistan

[c]Ghulam Ishaq Khan Institute of Engineering Sciences and Technology Topi, Khyber Pakhtunkhwa Pakistan

[d]Chemistry Department, Collage of Science, King Saud University, P. O. Box 2455, Riyadh-22451, Saudi Arabia

**Email: junaidkhan.nanotech@gmail.com

Figure S1: The X-ray Photoelectron Spectroscopy (XPS) analysis of bi-linker MOF.

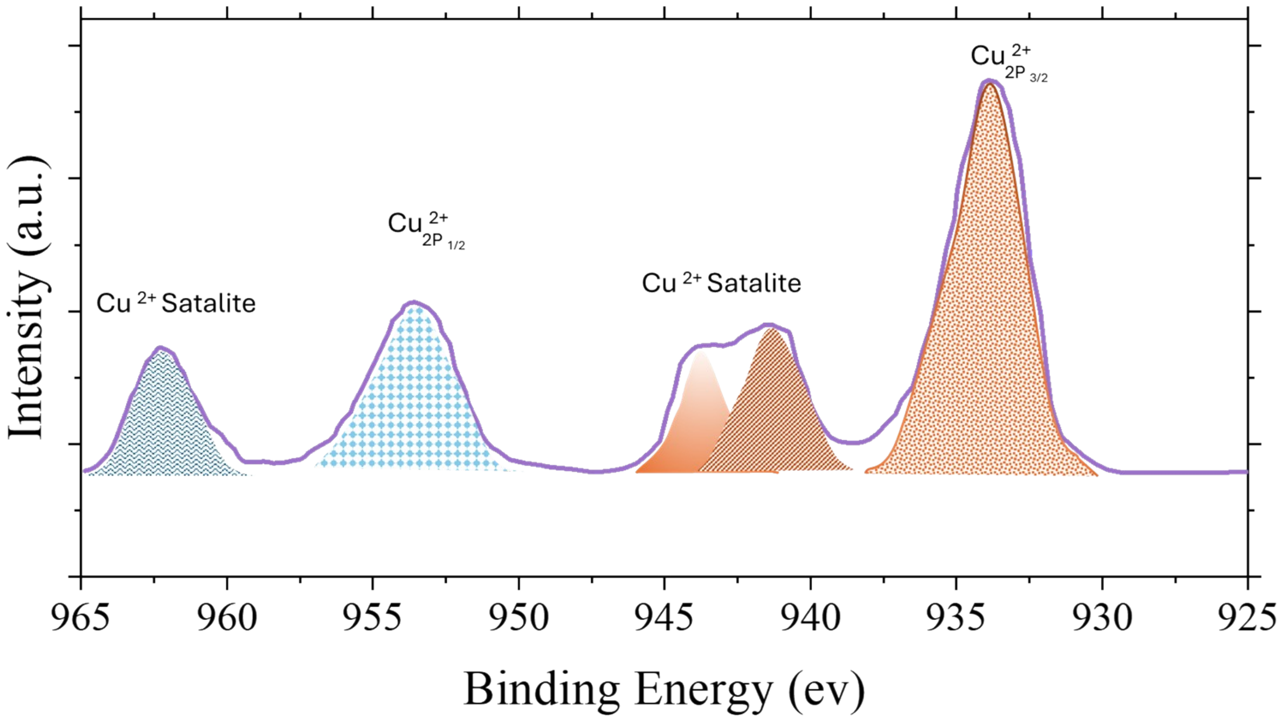

Figure S2: The N_2 Isotherms of all the samples.

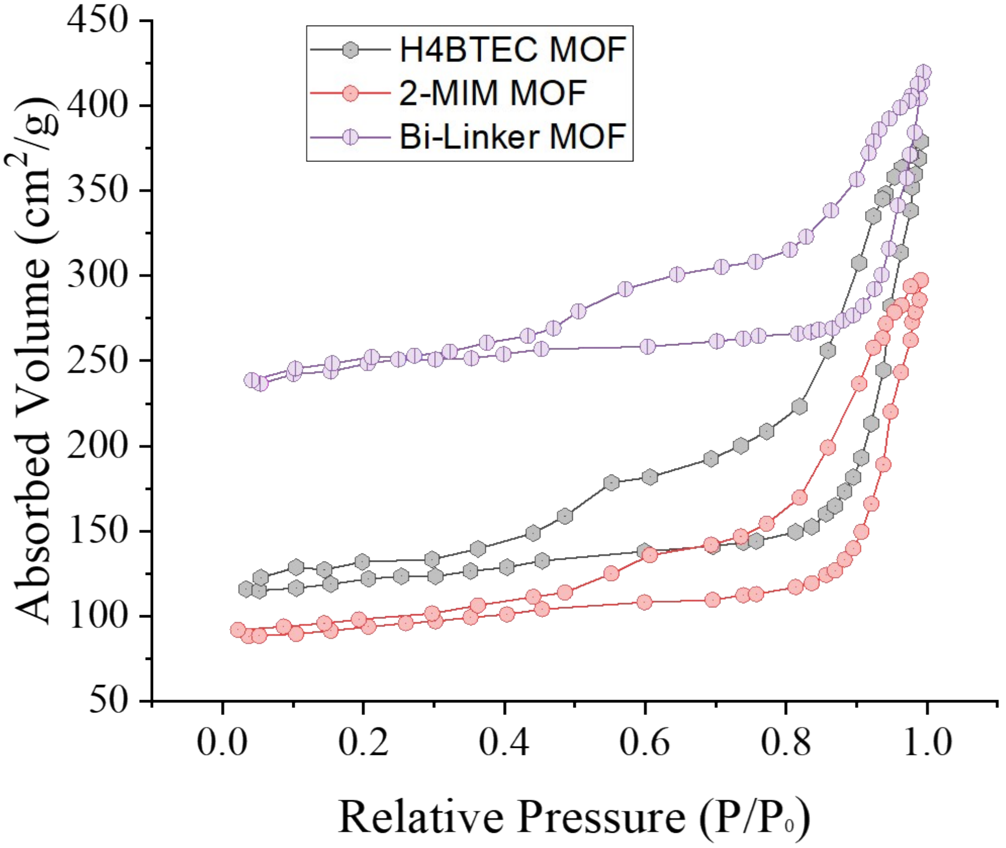

Figure S3: HER and OER curves of the bilinker MOF sample before and after the stability test.

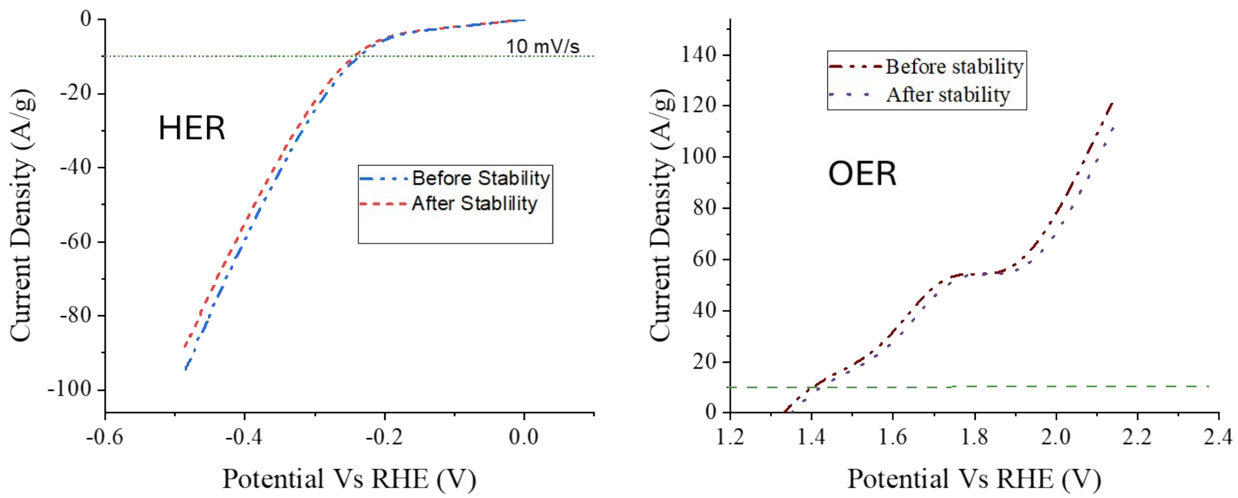

Supplement: RA-015-D5RA06407D-s001 [file RA-015-D5RA06407D-s001.pdf]
